# Long-term effects of goal disturbance and adjustment on well-being in cancer patients

**DOI:** 10.1007/s11136-015-1139-8

**Published:** 2015-10-07

**Authors:** Moniek Janse, Mirjam A. G. Sprangers, Adelita V. Ranchor, Joke Fleer

**Affiliations:** Department of Health Psychology, University Medical Center Groningen, University of Groningen, A. Deusinglaan 1, 9713 AV Groningen, The Netherlands; Department of Medical Psychology, Academic Medical Center Amsterdam, University of Amsterdam, Meibergdreef 15, 1105 AZ Amsterdam Zuidoost, The Netherlands

**Keywords:** Goal disturbance, Goal adjustment, Well-being, Cancer, Oncology

## Abstract

**Purpose:**

To investigate the impact of personal goal disturbance after cancer diagnosis on well-being over time, and a possible moderating role of goal adjustment tendencies and actual goal adjustment strategies.

**Methods:**

Participants (*n* = 186) were interviewed three times: within a month, 7 months (treatment period), and 18 months (follow-up period) after being diagnosed with colorectal cancer. Participants were asked to freely mention three to ten personal goals. Goal disturbance was assessed by the patients’ ratings of the amount of hindrance experienced in goal achievement. Goal adjustment tendencies were assessed using the Goal Disengagement and Re-engagement Scale and actual goal adjustment (i.e. goal flexibility) by the number of goal adjustment strategies used. Outcome measures were overall quality of life and emotional functioning, assessed with the cancer-specific EORTC QLQ-C30.

**Results:**

Hierarchical regression analyses showed that goal disturbance predicted well-being over both the treatment and the follow-up period. Additionally, the negative effect of goal disturbance on well-being was less for patients who scored higher on goal disengagement and not significant for patients who were more flexible in their use of actual goal adjustment strategies.

**Conclusions:**

The present study is the first to test the theoretical assumption that goal adjustment is beneficial after goal disturbance. Whereas these findings need to be confirmed in future research, the possibly beneficial role of goal disengagement and actual goal adjustment strategies can be used for psychological interventions.

## Introduction

Goals, their pursuit and achievement, are important as they give meaning and direction to people’s lives [1, 2]. Evidence from cross-sectional studies shows that the diagnosis of a severe illness such as cancer can lead to disturbances in goal pursuit, and that such disturbances are related to poorer well-being [3–5]. Whether goal disturbance continues to impact well-being over time, however, and what may moderate this impact, is still unknown. Theory assumes that when goal disturbance occurs, people need to adjust their goals to what is attainable to maintain acceptable levels of well-being (e.g. [6]). Yet, whether goal adjustment moderates the relation between goal disturbance and well-being over time has not been investigated. Hence, the current study will be the first to longitudinally investigate the predictive value of goal disturbance after cancer on well-being and test the theoretical assumption of a moderating role of goal adjustment. What is more, in addition to using the conventional operationalization of goal adjustment that assesses how people believe they will adjust their goals, the present study will also apply a novel approach assessing how people actually adjust their goals.

After a colorectal cancer diagnosis, physical problems could lead to difficulties in attaining goals and frequent hospital visits may leave less time in which goals can be pursued [5]. In general, goal disturbance was found to decline over time in people diagnosed with all stages of cancer [7, 8]. Yet there are indications that up to 18 months post-diagnosis, patients still report more health-related barriers to goal pursuit than healthy controls [8], which could thus affect well-being over that same period as well. Research is therefore needed investigating the long-term adverse effect of goal disturbance on well-being.

Even though it can be expected that almost all cancer patients experience some degree of goal disturbance after diagnosis, a large variability in well-being in patients remains. The ability to adjust disturbed goals may thus play a role in explaining the variation in well-being. Previous research on goal regulation in the context of health and illness, using the dual-process model, indeed showed that patients’ ability to flexibly adjust personal goals lessened the impact of the illness or health problem on psychological well-being (e.g. [9, 10]). To date, studies empirically investigating goal adjustment in people with cancer have focused almost exclusively on goal adjustment tendencies [4, 11–14]. Goal adjustment tendencies, or capacities, often refer to the ease with which one believes to be able to disengage from disturbed goals and re-engage in new attainable ones and are most commonly measured by the Goal Disengagement and Re-engagement Scale (GDRS) [15]. The tendencies can be assessed in general or in reference to specific situations, such as adjustment to cancer. It was commonly found that goal re-engagement was related to better well-being, but goal disengagement was not [4, 11, 13, 14, 16]. We therefore hypothesize that higher dispositional re-engagement may help patients maintain well-being when facing goal disturbance due to cancer.

How people believe they will adjust may not necessarily reflect how they actually adjust their goals. It is still unknown how goals really change or remain the same over time, and how this relates to well-being. Indeed, lately there have been repeated calls for long-term studies of actual goal adjustment with which to extend and complement goal research (e.g. [14, 17, 18]).

Four theories could be said to form the basis of goal adjustment: *the dual*-*process model of assimilative and accommodative coping* (e.g. [19, 20]), *the model of selection, optimization, and compensation* (*SOC*, e.g. [21–23]), *the life*-*span theory of control* (e.g. [24–26]), and *control theory* (e.g. [1, 2]). These theories mention several specific strategies people may use when adjusting their goals. The use of these strategies can be determined by systematically investigating personal goals over time [27], as all strategies imply a change in, or stability of, a person’s goals. Investigating the use of goal adjustment strategies can therefore serve as a measure for actual goal adjustment. Six beneficial adjustment strategies were deducted from the literature: *Shift priorities across life domains, Scale back goals in the same life domain, Scale up goals in the same life domain, Give up effort but remain committed/Put goals on hold*, *Form shorter*-*term goals* and *Form longer*-*term goals* [2, 6, 20, 26, 28, 29]. Their use is thought to be beneficial as they imply the continued engagement in important and attainable goals [6], but this has not yet been empirically examined.

Being capable of using a repertoire of adaptive goal adjustment strategies, instead of no or only one preferred strategy, has been suggested to benefit well-being [30, 31]. Flexibly deploying adjustment strategies enables people to respond to changing situations. Consequently, the more adjustment strategies are used after goals have been disturbed, the more this may help patients to maintain well-being. The flexible use of goal adjustment strategies will therefore be operationalized as the number of actual adjustment strategies used. The potentially beneficial role of goal adjustment within the relation between goal disturbance and well-being can thus be investigated. However, some strategies may be more beneficial than others. As the relations between the separate adjustment strategies and well-being are yet unknown, these will be examined as well.

Although higher dispositional re-engagement capacities and the use of more goal adjustment strategies are in general thought to be beneficial for well-being, the extent of this effect may depend on the specific situation in which they are required or used. It may be necessary to re-engage in new goals or use many adjustment strategies during the first chaotic months after diagnosis (i.e. the treatment period), as coming to terms with the initial diagnosis and consequences of the illness may require the adjustment of many goals (early loss-based selection) [8]. When facing (early) survivorship or end-of-life during the phase thereafter (i.e. the follow-up period) [32, 33], adopting new goals or adjusting goals may be somewhat less urgent, as adaptation to the most sudden life changes has already taken place. Higher goal re-engagement capacities and the use of more goal adjustment strategies may thus be more beneficial during the treatment period than the follow-up period.

**Fig. 1:**
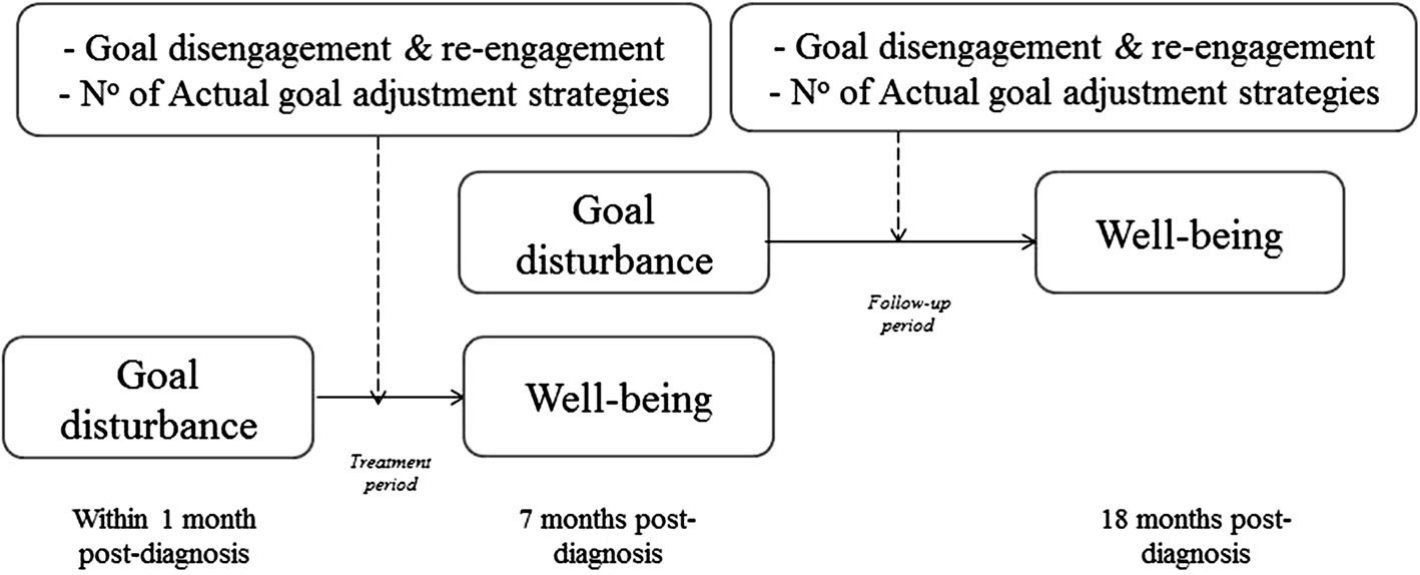
Research design

In sum, the present study aims to answer the following research questions: (1) does goal disturbance within a month post-diagnosis predict well-being 7 months post-diagnosis (i.e. the treatment period) and does goal disturbance 7 months post-diagnosis predict well-being 18 months post-diagnosis (i.e. the follow-up period), and (2) does goal adjustment (i.e. goal adjustment tendencies and number of beneficial actual goal adjustment strategies used) moderate the relation between goal disturbance and well-being over the treatment period and the follow-up period? With respect to these research questions, the following hypotheses were formulated: (1) goal disturbance predicts well-being over both periods, with more goal disturbance leading to poorer well-being, and (2) a higher tendency to re-engage and the flexible use of more actual goal adjustment strategies will buffer the adverse effect of goal disturbance on well-being. It is assumed that this effect will be visible during both the treatment and follow-up periods, but will be more pronounced during the treatment period. In addition, although we expect all goal adjustment strategies to be beneficial for well-being, due to the novelty of the use of actual goal adjustment strategies, each goal adjustment strategy will also be separately analysed as a moderator in the relation between goal disturbance and well-being. Figure 1 depicts the research design guiding this study.

## Methods

The current paper used the same database that was used for a previous paper in which the construction of goal strategies was reported [34].

### Design and participants

Between September 2011 and March 2013, all newly diagnosed people with medically confirmed colorectal cancer from four participating hospitals in the Netherlands were invited to participate. Exclusion criteria were: insufficient understanding of the Dutch language, drugs- and/or alcohol-related problems, a cognitive impairment or psychiatric disorder, and being under the age of 18. There were three assessment points: within 1 month post-diagnosis, 7, and 18 months post-diagnosis. The treatment period was defined as the period between 1 month and 7 months post-diagnosis, and the follow-up period between 7 and 18 months post-diagnosis. The study was approved by the medical ethical committee of a university medical centre in the Netherlands, and all patients provided informed consent.

### Procedure

A member of the hospital staff explained the study to eligible patients and handed them an information package. The package contained an information letter, an informed consent form, and a prepaid envelope. Respondents were asked to read the information at home and return the informed consent form if they agreed to participate. After informed consent was received, the researchers assigned a trained interviewer to the respondent to conduct all three assessments at a place of the patients’ choice, mostly their homes.

### Measures

#### Demographic characteristics

During the first assessment, within a month post-diagnosis, information was collected concerning respondents’ age and gender.

#### Goal disturbance

At all three assessments, respondents were asked to list three to ten personal goals, explained to them as projects they were currently working on, activities they were busy with, or plans they wanted to achieve (based on e.g. [1, 8]). Goal disturbance was assessed with a single item for each goal: To which degree does your illness hinder you in achieving your goal? Answers were given on a 10-point Likert scale ranging from 1 (not at all) to 10 (very) (based on e.g. [1, 8]). Mean goal disturbance scores from all goals at each assessment were calculated per respondent.

#### Goal adjustment

##### Goal adjustment tendencies

To assess the ease with which someone believes to be able to disengage from unattainable goals and re-engage in new, meaningful goals, respondents completed the Goal Disengagement and Re-engagement Scale (GDRS, [15]). This questionnaire consists of 10 items, 4 measuring disengagement and 6 measuring re-engagement, to be answered on a 5-point scale ranging from 1 ‘almost never true’ to 5 ‘almost always true’. Goal adjustment tendencies were assessed one and 7 months following diagnosis. Cronbach’s alpha was .66 for goal disengagement and .91 for goal re-engagement at one month post-diagnosis and .76 for goal disengagement and .91 for goal re-engagement 7 months post-diagnosis.

##### Goal adjustment strategies

In a previous paper, we calculated the use of six goal adjustment strategies (*Shift priorities across domains, Scale back goals in same life domain, Scale up goals in same life domain, Give up effort but remain committed/Put goals on hold, Form shorter*-*term goals* and *Form longer*-*term goals*) for the two periods studied (i.e. the treatment and follow-up periods) in the current sample [34]. In that paper, the calculation of the strategies is also explained in detail. The current paper builds upon the previous study by investigating the moderating role of the goal adjustment strategies in the relation between goal disturbance and well-being. To be able to calculate the use of the actual goal adjustment strategies, goal characteristics over time were used. Goal characteristics entail *goal content* (life domain: physical, psychological, social, achievement, leisure, and level of abstraction: very abstract—very concrete) and g*oal structure* (importance, attainability, effort, and temporal range) (based on e.g. [1, 8]). Scoring formulas were developed for each strategy using those characteristics over time important for defining their use. For instance, for the strategy *Scale back goals in the same life domain*, the characteristics life domain and level of abstraction were used, making it possible to determine whether goals within the same life domain decreased in level of abstraction over time (for the complete operationalization of each of the strategies, see [34]). For the purpose of the current study, the flexible use of actual goal adjustment strategies was assessed by the total number of goal adjustment strategies used for each period (i.e. 0 = no goal adjustment strategies used and 6 = 6 goal adjustment strategies used).

#### Well-being

##### Quality of life

QoL was assessed using the Global health status/QoL subscale of the Quality of Life Questionnaire-Core 30 (QLQ-C30) version 3.0 [35]. This questionnaire assesses the quality of life of cancer patients and is developed by The European Organization for Research and Treatment of Cancer (EORTC). The items were: ‘How would you rate your overall health during the past week’ and ‘How would you rate your overall quality of life during the past week?’ Patients answered these items on a 7-point Likert scale ranging from ‘very bad’ to ‘excellent’.

##### Emotional functioning

Emotional functioning was assessed using the emotional functioning subscale of the EORTC QLQ-C30 [35]. This subscale consisted out of four items, answered on a scale from 1 = not at all to 4 = very much. An example item is: ‘Did you worry?’

Following the EORTC QLQ-C30 scoring guidelines, the raw scores of both scales were standardized to a scale from 0 to 100 using a linear transformation, with higher scores indicating better QoL or emotional functioning.

### Data analysis

First, descriptive statistics and repeated-measures analyses with time as a within-subjects factor were performed to examine changes in mean levels of goal disturbance, goal adjustment, and well-being within 1 month, 7 and 18 months post-diagnosis. Second, due to the novelty of the method of assessing actual goal adjustment, correlations between the commonly used goal disengagement and re-engagement tendencies and the number of actual goal adjustment strategies were investigated. Then, four separate hierarchical regression analyses were performed. First, the predictive value of goal disturbance within 1 month post-diagnosis on QoL 7 months post-diagnosis was assessed, and the moderating role of goal adjustment tendencies at diagnosis and actual goal adjustment during the treatment period. Second, this same analysis was performed with emotional functioning as the outcome measure. Third, the predictive value of goal disturbance 7 months post-diagnosis on QoL 18 months post-diagnosis was assessed, and the moderating role of goal adjustment tendencies 7 months post-diagnosis and actual goal adjustment during the follow-up period. Finally, this same analysis was performed with emotional functioning as the outcome measure. We performed Pearson correlations to check whether we had to control for socio-demographic variables. Age correlated significantly with goal disturbance, goal adjustment tendencies, and both well-being measures and was entered in step one of the analyses. To investigate the possible interaction effects and to increase interpretability of the parameter estimates, the independent variable and potential moderators were centred, meaning that from each data point, the mean was subtracted. The centred variables of goal disturbance (step 2), goal disengagement, and re-engagement (step 3) and the use of goal adjustment strategies (step 4) were entered into the model. These variables were then used to create the interaction variables, which were entered into step five of the regression analyses. Additionally, each goal adjustment strategy was also separately investigated as a moderator. When the interaction was significant, we preformed post hoc tests by comparing the simple slopes for 1 SD above and below the mean of the moderator to investigate the direction of the relationship. Results were tested two-sided, and a *p* value of <.05 was considered significant throughout. Statistical Package for Social Sciences (IBM SPSS) version 22.0 for Windows was used for the statistical analysis.

## Results

### Patients

During the inclusion period, 622 eligible patients were identified. Of these patients, 46 were already engaged in other studies and could not be approached, and 64 patients were not offered the information due to procedural errors in the hospitals. For 15 patients, why they did not receive the information was not documented. Of the 497 patients who were offered the information regarding the study, 380 patients accepted this and 228 signed informed consent (response rate: 45.9 %). Over time, 219 patients completed the first assessment, 201 completed the second assessment, and 186 completed all three assessments (dropout rate of 15.1 %). Of the 186 respondents, 39.2 % were female, and the mean age was 64.2 years (for the complete flowchart, see [34]).

### Well-being, goal disturbance, and goal adjustment over time

Over time, respondents reported a significantly improved QoL (*F* = 11.3, *p* < .001) and better emotional functioning (*F* = 26.53, *p* < .001). Additionally, they reported significantly less goal disturbance from 1 to 18 months post-diagnosis (*F* = 21.85, *p* < .001). Mean scores on the GDRS subscales remained stable, and patients used more goal adjustment strategies during the follow-up period compared to the treatment period (*t* = −2.78, *p* = .01) (see Table 1).

**Table 1:** Data for QoL, emotional functioning, goal disturbance, and adjustment over time (*n* = 186)

Variable	*M* (SD) Time 1: within 1 month post-diagnosis	*M* (SD) Time 2: 7 months post-diagnosis	*M* (SD) Time 3: 18 months post-diagnosis	*F* (*p*)^a^
Quality of life (EORTC)	72.8 (20.8)	76.6 (19.1)	80.1 (17.9)	11.3 (<.001)
Emotional functioning (EORTC)	75.6 (20.1)	83.1 (19.2)	85.1 (19.2)	26.53 (<.001)
Goal disturbance	4.7 (2.4)	4.1 (2.7)	3.3 (2.6)	21.85 (<.001)
Goal adjustment tendencies				*t* (*p*)^b^
Goal disengagement	11.9 (3.1)	11.9 (3.1)		−.20 (.84)
Goal re-engagement	21.2 (4.6)	21.3 (4.3)		−.42 (.68)
	*Period 1* ^c^	*Period 2* ^d^		*t* (*p*)
No. of goal adjustment strategies	1.3 (.9)	1.6 (1.0)		−2.78 (.01)

^a^Repeated-measures GLM with 3 factors, factor = time

^b^Paired sample *t* test

^c^Period 1 = between 1 and 7 months post-diagnosis

^d^Period 2 = between 7 and 18 months post-diagnosis

During the treatment period, 80.6 % of respondents used a goal adjustment strategy, while during the follow-up period, 87. 6 % used a strategy. Respondents who used a strategy during the treatment period mostly used one strategy (44.6 %). Twenty-six percent used two strategies, 7 % used three strategies, and 2.7 % used four strategies. During the follow-up period, 38.2 % used one strategy, 34.9 % used two strategies, 11.3 % used three strategies, and 3.2 % used four strategies.

### Goal adjustment measures

Higher scores on goal disengagement within a month post-diagnosis were found to be significantly correlated with the use of less actual goal adjustment strategies during the treatment period (*r* = −.17, *p* = .02). Higher goal re-engagement scores within a month post-diagnosis were significantly correlated with the use of more goal adjustment strategies during the follow-up period (*r* = .16, *p* = .03).

### Does goal disturbance predict well-being over time?

#### Treatment period

The final model of the hierarchical regression analyses for predicting well-being 7 months post-diagnosis revealed that age and goal disturbance significantly predicted QoL with younger age and higher goal disturbance being associated with decreased QoL (see Table 2). With respect to emotional functioning, the final model showed that higher goal disturbance significantly predicted lower emotional functioning.

**Table 2:** Hierarchical regression analyses predicting well-being 7 months post-diagnosis (Time 2) controlling for age (step 1) and entering goal disturbance (step 2), goal disengagement, and goal re-engagement (step 3) within a month post-diagnosis (Time 1) and no. of goal adjustment strategies (step 4) between 1 and 7 months post-diagnosis (Period 1). Interaction terms were entered in step 5

	Step 1		Step 2		Step 3		Step 4		Step 5	
	*b* (SE)	Beta	*b* (SE)	Beta	*b* (SE)	Beta	*b* (SE)	Beta	*b* (SE)	Beta
*Quality of life Time 2*										
Age	.40 (.13)	.23**	.28 (.13)	.16*	.27 (.14)	.15	.27 (.14)	.15	.30 (14)	.17*
Goal disturbance Time 1	–		−2.16 (.58)	−.27**	−2.06 (.58)	−.26**	−2.06 (.58)	−.26**	−2.22 (.59)	−.28**
Goal disengagement Time 1	–		–		.48 (.48)	.08	.53 (.49)	.09	.51 (.49)	.08
Goal re-engagement Time 1	–		–		.16 (.33)	.04	.15 (.33)	.04	.13 (.33)	.03
No. of goal adjustment strategies Period 1	–		–		–		1.02 (1.45)	.05	1.3 (1.5)	.06
Goal disturbance × goal disengagement	–		–		–		–		.44 (.22)	.16*
Goal disturbance × goal re-engagement	–		–		–		–		−.17 (.12)	−.10
Goal disturbance × No. of goal adjustment strategies	–		–		–		–		.67 (.62)	.08
Δ*R* ^2a^	.05**		.07**		.01		.00		.03 total = .16	
*Emotional functioning Time 2*										
Age	.37 (.13)	.21**	.26 (.13)	.15*	.22 (.14)	.12	.22 (.14)	.12	.25 (.14)	.15
Goal disturbance Time 1			−1.91 (.6)	−.24**	−1.78 (.58)	−.23**	−1.78 (.58)	−.23**	−1.82 (.58)	−.23**
Goal disengagement Time 1					.97 (.48)	.16*	.9 (.49)	.15	.9 (.49)	.15
Goal re-engagement Time 1					.07 (.32)	.02	.08 (.33)	.02	.04 (.32)	.01
No. of goal adjustment strategies Period 1							−1.22 (1.44)	−.06	−1.01 (1.4)	−.05
Goal disturbance × goal disengagement									.35 (.22)	.13
Goal disturbance × goal re-engagement									−.08 (.12)	−.05
Goal disturbance × No. of goal adjustment strategies									1.26 (.61)	.15*
Δ*R* ^2^	.04**		.06**		.03		.00		.03 total = .16	

* *p* < .05; ** *p* < .01

^a^Percentage of variance explained by the model

#### Follow-up period

During the follow-up period (see Table 3), the final models showed that higher goal disturbance significantly predicted lower QoL and lower emotional functioning.

**Table 3:** Hierarchical regression analyses predicting well-being 18 months post-diagnosis (Time 3) controlling for age (step 1) and entering goal disturbance (step 2), goal disengagement, and goal re-engagement (step 3) 7 months post-diagnosis (Time 2) and no. of goal adjustment strategies (step 4) between 7 and 18 months post-diagnosis (Period 2). Interaction terms were entered in step 5

	Step 1		Step 2		Step 3		Step 4		Step 5	
	*b* (SE)	Beta	*b* (SE)	Beta	*b* (SE)	Beta	*b* (SE)	Beta	*b* (SE)	Beta
*Quality of life Time 3*										
Age	−.03 (.12)	−.02	−.11 (.12)	−.07	−.09 (.13)	−.06	−.13 (.13)	−.08	−.12 (.13)	−.07
Goal disturbance Time 2	−		−2.02 (.49)	−.3**	−1.87 (.5)	−.28**	−1.99 (.49)	−.29**	−1.96 (.5)	−.29**
Goal disengagement Time 2	–		–		.25 (.45)	.04	.37 (.45)	.06	.32 (.46)	.05
Goal re-engagement Time 2	–		–		.45 (.33)	.11	.34 (.33)	.08	.34 (.33)	.08
No. of goal adjustment strategies Period 2	–		–		–		3.01 (1.34)	.16*	2.96 (1.34)	.16*
Goal disturbance × goal disengagement	–		–		–		–		−.02 (.16)	−.01
Goal disturbance × goal re-engagement	–		–		–		–		.12 (.11)	.08
Goal disturbance × No. of goal adjustment strategies	–		–		–		–		−.17 (.52)	−.02
ΔR^2a^	.00		.09**		.02		.03*		.01 total = .15	
*Emotional functioning Time 3*										
Age	.26 (.13)	.15*	.2 (.13)	.11	.16 (.14)	.09	.13 (.14)	.08	.15 (.14)	.09
Goal disturbance Time 2	–		−1.46 (.53)	−.2**	−1.24 (.53)	−.17*	−1.34 (.53)	−.19*	−1.23 (.54)	−.18*
Goal disengagement Time 2	−		−		.87 (.49)	.14	.97 (.49)	.16	.87 (.50)	.14
Goal re-engagement Time 2	−		−		.18 (.35)	.04	.1 (.36)	.02	.07 (.36)	.02
No. of goal adjustment strategies Period 2	−		−		−		2.36 (1.47)	.12	2.17 (1.48)	.11
Goal disturbance × goal disengagement	−		−		−		−		−.07 (.18)	−.03
Goal disturbance × goal re-engagement	–		–		–		–		.22 (.12)	.14
Goal disturbance × No. of goal adjustment strategies	–		–		–		–		−.23 (.56)	−.03
Δ*R* ^2^	.02*		.04**		.02		.01		.02 total = .11	

* *p* < .05; ** *p* < .01

^a^Percentage of variance explained by the model

### Do goal adjustment tendencies and number of actual goal adjustment strategies used moderate the relation between goal disturbance and well-being?

#### Treatment period

**Fig. 2:**
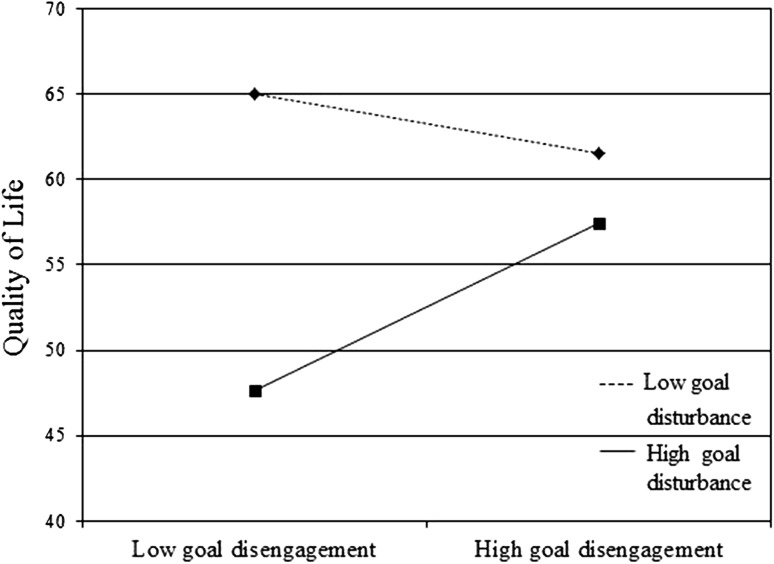
Goal disengagement as a moderator of Quality of Life

Three potential interactions (i.e. goal disturbance × goal disengagement, goal disturbance × goal re-engagement, and goal disturbance × number of goal adjustment strategies) were entered to first predict QoL. Table 2 shows that only goal disengagement significantly moderated the relation between goal disturbance and QoL. To illustrate and further explore this significant interaction, we calculated and plotted the regression slopes for patients at two levels of goal disturbance: high (+1 SD) and low (−1 SD). These analyses showed that although goal disturbance had an overall negative impact on QoL, this effect was greater for patients who scored low on goal disengagement (*b* = −3.27, *p* ≤ .001) than those who scored high on disengagement (*b* = −1.68, *p* = .03) (see Fig. 2). Investigating each goal adjustment strategy independently as a potential moderator showed no significant results (data not shown).

**Fig. 3:**
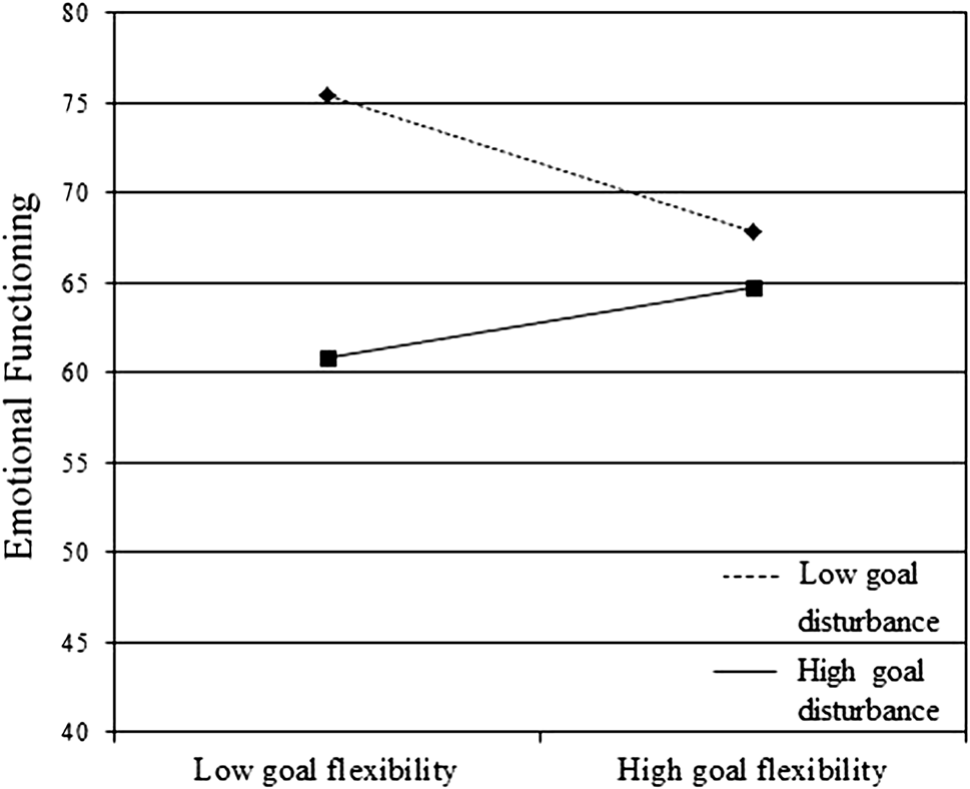
Use of actual goal adjustment strategies (i.e. goal flexibility) as a moderator of Emotional Functioning

With respect to emotional functioning, only the number of goal adjustment strategies used was a significant moderator (see Table 2). Post hoc analyses showed that the negative association between levels of goal disturbance and emotional functioning was significant when goal flexibility was low (*b* = −2.82, *p* ≤ .001), but not when goal flexibility was high (*b* = −1.35, *p* = .10) (see Fig. 3). Investigating each goal adjustment strategy independently as a moderator showed that the use of the strategy *Scale up goals in the same life domain* moderated the relation between goal disturbance and emotional functioning (*b* = 3.42, SE = 1.45, *t* = 2.36, *p* = .02), suggesting that the use of this strategy buffered the adverse effect of goal disturbance on emotional functioning (data not shown).

#### Follow-up period

None of the three potential interactions entered in step 5 significantly moderated the relation between goal disturbance and QoL, or goal disturbance and emotional functioning. The results of the analyses investigating adjustment strategies independently as a moderator showed no significant results (data not shown).

## Discussion

The present study set out to longitudinally investigate the theoretical assumptions that goal disturbance negatively impacts well-being, and that goal adjustment may reduce the adverse effect of goal disturbance on well-being. The results show that, in line with our hypothesis, higher levels of goal disturbance indeed predicted lower levels of well-being between 1 and 7 months post-diagnosis (i.e. the treatment period), as well as between 7 and 18 months post-diagnosis (i.e. the follow-up period). Additionally, also in line with our hypothesis, we found the first indications that a higher tendency to disengage and the flexible use of more actual goal adjustment strategies may buffer the adverse effect of goal disturbance on well-being during the treatment period.

The findings of the current study are in agreement with previous cross-sectional studies demonstrating the adverse effect of goal disturbance on well-being [3–5]. Moreover, they show that goal disturbance is a consistent predictor of quality of life (QoL), as well as emotional functioning, up to 18 months post-diagnosis. These results stress the importance of goal disturbance after cancer in determining well-being. However, it could be suggested that goal disturbance was not particularly high at any assessment point (i.e. the maximum mean score was 4.7 (SD 2.4) on a scale from 1 to 10 at the first assessment). As the first assessment point was within 1 month after cancer diagnosis, patients could already have started adjusting their goals in the time between diagnosis and the first assessment, in keeping with the model of immediate loss-based selection [8, 21]. Also, according to theories of lifespan development, higher age is related to decreasing opportunities for goal achievement. People can anticipate this by adjusting their goals to match decreasing resources [26]. As our older sample may have already started adjusting their goals, they could have experienced lower levels of goal disturbance.

With respect to the moderating role of goal adjustment tendencies, during the treatment period, the negative effect of goal disturbance on QoL was less for patients who scored higher on goal disengagement. These findings are in line with our hypothesis and suggest that being capable of disengaging from goals when experiencing goal disturbance can buffer the adverse effect of goal disturbance and help patients maintain well-being. Notably, prior (mostly cross-sectional) research in people with cancer found beneficial effects only for goal re-engagement and not for goal disengagement [4, 13, 14, 16]. Yet, these studies focused on cancer survivors who were assessed at various times since diagnosis, i.e. from 10 months [16] to 7 years [13, 14]. It may thus be that goal disengagement is particularly adaptive in the treatment period during the first months following cancer diagnosis. During these hectic months, patients may need to (temporarily) let go of their previously important goals to be able to focus on treatment and coming to terms with their cancer diagnosis. Still, the effects of goal disengagement were modest, and future research is needed to confirm these findings.

Furthermore, our results suggest that high goal flexibility (i.e. the use of more actual goal adjustment strategies) could be beneficial for emotional functioning when experiencing goal disturbance, as patients who were flexible did not report significantly lower emotional functioning when experiencing goal disturbance, while patients who were not flexible in adjusting their goals did. This finding is similar with respect to goal disengagement, suggesting that goal adjustment becomes important for maintaining well-being once goals are disturbed. Indeed, coping or adjustment flexibility refers specifically to the capacity to deploy various strategies in reaction to stressful life changes [30, 31]. Again, we only found a significant interaction effect only during the treatment period. During the first months of diagnosis and treatment, more choices and considerations might be necessary to deal with goal disturbance. While it is in general thought that people adjust their goals throughout their lives, this may be extra important following a cancer diagnosis. During the year thereafter, it may be less urgent to react to sudden goal disturbances, but more to permanently changed life circumstances. Goal adjustment may then again be part of natural and developmental adjustment and have less added value. Also, we did not find a relation with quality of life, but only with emotional functioning. More research is needed before we can make firm conclusions concerning the role of goal adjustment in well-being after cancer.

An additional finding was a main effect of goal flexibility on QoL during the follow-up period, indicating that those who used more actual goal adjustment strategies between 7 and 18 months post-diagnosis, reported higher QoL 18 months post-diagnosis. It has been suggested that in the period following treatment, dealing with the emotional consequences of cancer becomes more central to patients [33]. They need to come to terms with possible long-term effects of the illness, but also with the realizations of the finiteness of life or even with the fact that limited time is left [36, 37]. Such changes in life perspective may be accompanied by changes in goals. Under such circumstances, it may be beneficial to use more adjustment strategies. Yet, additional studies are needed to investigate these findings further.

Due to the novelty of the method to assess actual goal adjustment strategies, we briefly examined the relations between the new method of assessing actual goal adjustment and the method that has been most commonly used to assess goal adjustment, the goal adjustment tendencies (i.e. goal disengagement and goal re-engagement). We found that patients who scored higher on the tendency to disengage used less goal adjustment strategies during the treatment period. This finding may seem counter-intuitive, as people who are better at disengaging may be more likely to use adjustment strategies in which this is important. Currently, we do not have the appropriate data to further explore these findings. However, as goal disengagement is assessed as a general tendency, it could be that people may believe that they will disengage from disturbed goals, but that in the specific situation of goal disturbance due to a major life event like cancer, they find it harder to actually do so. Thus, they may react differently in these specific circumstances. Another finding was that patients who scored high on goal re-engagement measured within a month after diagnosis, tended to use more goal adjustment strategies during the follow-up period. It could be that people, who believe to be capable of re-engaging, only get the chance to do so during the follow-up period and use strategies involving re-engagement into new goals. However, these findings and interpretations should be investigated further.

The current study has several strengths, namely its large sample size, longitudinal design and novel approach towards assessing actual goal adjustment. The validity of the method to investigate the use of the actual goal adjustment strategies is not established, and this could be seen as a limitation even though the method was tested in an earlier study [27].

Findings of the present study provide directions for future research. As the effect of goal disturbance and adjustment on the two different well-being measures (i.e. QoL and emotional functioning) differed, more research is needed to investigate the mechanisms behind the different goal adjustment measures and how they relate to well-being measures. In addition, as touched upon earlier, age could cause differences in goal disturbance and adjustment. It could thus be relevant to investigate goal disturbance and adjustment, as well as their impact on well-being, in younger patient samples. Furthermore, as the current study found support for the long-term adverse effect of goal disturbance, it seems especially important to continue to study how goal adjustment may help patients maintain well-being. Considering that this study has made only the first steps towards testing existing theories on goal disturbance and adjustment, and the explained variance in our models remained modest, future research is needed to replicate and extend these findings.

With respect to the clinical implications, we found indications that both higher reported general goal disengagement capacities and especially the use of more actual goal adjustment strategies could be beneficial after goal disturbance. Goal disengagement, however, assesses a general and stable trait and might therefore be difficult to intervene upon. On the other hand, the novel method of adjustment strategies provides clear suggestions of concrete actions that can be practiced in psychological interventions. Also, when offering interventions focusing on goal adjustment, the current results suggest that it seems to be important to do so within the first months following diagnosis. The study therefore adds new pieces of knowledge on what may be beneficial for patients’ well-being at specific time points after a cancer diagnosis.

In sum, the present study has made a step in advancing the field of goal research by answering to the call for longitudinal studies on goal disturbance, actual goal adjustment, and well-being. Findings highlight the relevance of focusing on what actually happens to goals after goal disturbance due to cancer in specific periods after diagnosis, and how this influences well-being.
